# Subjective well-being patterns in older men and women without someone to confide in: a latent class analysis approach

**DOI:** 10.3389/fpubh.2023.1286627

**Published:** 2024-01-05

**Authors:** Dijuan Meng, Chang Sun

**Affiliations:** School of Nursing, Nanjing University of Chinese Medicine, Nanjing, China

**Keywords:** older adults, confidants, subjective well-being, latent class analysis, CLHLS

## Abstract

**Objective:**

This study aimed to identify the latent subtypes of subjective well-being (SWB) and associated factors in older adults without a confidant in China.

**Methods:**

The data came from the most recent (seventh) wave (2018) of the Chinese Longitudinal Healthy Longevity Survey (CLHLS). This cross-sectional study included 350 older adults who lacked a close confidant. We utilized latent class analysis and multiple logistic regression models to examine the latent SWB subtypes and associated factors.

**Results:**

Three distinct patterns of SWB were identified: the very low SWB class (32%), the medium-low SWB class (46%), and the low evaluative and high affective SWB class (22%). The results indicated that compared to the low evaluative and high affective SWB class, respondents who self-rated their health as not good, currently drank alcohol and rated their financial status as poor/very poor were more likely to be in the very low SWB class, while those who participated in social activities were less likely to be in the very low SWB class. Respondents who had limitations in instrumental activity of daily living (IADL) and rated their financial status as poor/very poor were more likely to be in the medium-low SWB class. However, gender did not affect SWB patterns.

**Conclusion:**

Our findings highlight awareness of the heterogeneity of SWB in older adults without close confidants and provide valuable information for the development of tailored intervention programs to improve their well-being.

## 1 Introduction

During the past decades, considerable achievements have been made in reducing mortality and increasing life expectancy in many countries (1, 2), and most people can expect to live longer than ever. However, whether older adults experience these added years in good health or with decreased physical or mental capacity has different implications for these individuals and for society. It has become the consensus that fostering healthy aging is the most cost-effective means to address the rising aging population (3). Healthy aging is defined by the World Health Organization (WHO) as the process of developing and maintaining functional ability that enables well-being in older age (4), and the ability to build and maintain relationships is proposed as one of the five functional areas that need to be optimized among older adults (3). An active social life and supportive social interactions serve as protective factors against a decline in well-being in late life (5).

Maslow’s hierarchy of needs theory suggests that establishing connections with others, intimacy and a sense of belonging are some of the most important human needs that must be fulfilled. According to socioemotional selectivity theory (6–8), although there is an age-related reduction in the size of older adults’ social networks, this occurs primarily in more peripheral relationships. As people age, they tend to restrict their social networks because they feel that the future is limited. They are likely to be motivated to preserve and improve their closest social ties, which helps to stabilize well-being in late life. The availability of trusted, close confidants is considered the “core” of older adults’ social ties. Considering the above analysis, it can be expected that close social ties and their benefits matter more for the well-being of older adults than novel or peripheral ties.

The “social buffering” hypothesis suggests that social support can improve adaptability and facilitate individuals’ long-term well-being (9). Studies have shown that the confidant relationships available to older adults are associated with higher well-being, better quality of life and lower depression and anxiety (10–14). In contrast, the lack of a close confidant is especially difficult for older adults. A study conducted in 16 European countries found that older respondents with no named confidants had the lowest level of well-being, highlighting the importance of identifying older people who have no confidants on whom to rely (10). Newton et al. (15) found that in a primary care setting, ill middle-aged and older adults without a close confidant tend to have higher levels of anxiety and depression. Likewise, in a sample of 2,670 community-dwelling older people residing in Quebec, Mechakra-Tahiri et al. found that confidant availability was negatively linked with depression in both older men and older women (13). However, gender differences may exist in confidant network types and the response to confidant availability or unavailability. Previous studies have found that being with or without a family or friend confidant is more strongly associated with depressive symptoms in women than in men (14, 16).

While existing research sheds light on the significance of close confidants to the well-being of older adults, there is an increasing awareness of the diverse and intricate factors influencing their subjective well-being (SWB). Sociodemographic variables extend beyond gender (14, 17), as mentioned previously, to include age (10, 14, 15), living arrangements (10), educational attainment (10, 14), marital status (14, 17), and so on. Health-related factors such as self-rated health (17, 18) and limitations in physical activity (19, 20) are thoroughly examined, alongside financial stability (14). The literature further underscores how the presence or absence of a confidant profoundly impacts older adults’ inclination to pursue mental health services and engage in psychological treatments (21, 22).

However, the majority of studies have focused on the association between the presence of confidants and a single dimension of well-being, frequently in relation to negative emotions such as depression and anxiety, and these studies are largely based within Western cultural settings. Nevertheless, subjective well-being (SWB), recognized as a justified proxy for an individual’s level of well-being (23), is inherently multidimensional, encompassing both evaluative and affective components (24, 25). Evaluative well-being refers to how people appraise the overall state of their own lives, while affective well-being focuses on the degree of positive or negative emotions and moods a person experiences. A happy life should include rare experiences of negative affect as well as high life satisfaction and frequent experiences of positive affect. Therefore, it seems meaningful to focus on the association between the availability of close confidants and older adults’ SWB from multiple domains. In addition, we should consider the heterogeneity of SWB in older adults without close confidants.

This study used latent class analysis (LCA) to identify the latent subtypes of SWB in older adults who had no confidant in China, a non-western culture. It also aimed to explore how these SWB patterns are associated with or influenced by their characteristics. A more detailed understanding of the profiles of different SWB classes can guide the development of targeted interventions. In addition, we hypothesized that there would be gender differences in patterns related to older adults’ SWB.

## 2 Materials and methods

### 2.1 Data and sample

The study adopts a cross-sectional methodology, utilizing data from the latest (seventh) wave (2018) of the Chinese Longitudinal Healthy Longevity Survey (CLHLS), a comprehensive nationwide prospective cohort study. The baseline survey was conducted in 1998; subsequent follow-up waves were performed in 2000, 2002, 2005, 2008, 2011, 2014, and 2018. The survey was initially launched to investigate factors that affected healthy longevity in humans and thus provided an excellent source for the research. This study was approved by the Ethics Committee of Peking University (IRB00001052-13074), and all participants and/or their families gave written informed consent.

Considering that our aim was to explore SWB patterns in older men and women who lacked a confidant, two inclusion criteria were imposed on the study sample. (a) Respondents should be ≥65 years old. Based on previous studies, people aged 106 and older were excluded due to insufficient information to validate these extremely high ages (26, 27). (b) Respondents answered that they had no one to confide in when needed. A total of 138 older men and 212 older women were included as the research sample for the present analyses (Supplementary Figure S1).

### 2.2 Measures

#### 2.2.1 Confidant availability

In the 2018 wave of the CLHLS, information on the availability of confidants was obtained through the question, “Is there someone with whom you can share your very private feelings and concerns?” The potential responses were spouse; son; daughter; daughter-in-law; son-in-law; grandchildren and their spouses; other relatives; friends/neighbors; social workers; housekeeper; nobody. Our analyses were restricted to the respondents who answered “nobody.”

#### 2.2.2 Subjective well-being

In this study, we used two measures that covered the evaluative and affective dimensions to examine respondents’ SWB patterns (28, 29). Evaluative SWB was assessed by a single question, “How do you feel about your present life?” Affective SWB was measured with five items. Two of them asked the respondents how often they “look[ed] on the bright side of things” and “[were] as happy as when they were young” in the previous week, which reflects the positive indicators of affective SWB. The other three items asked the respondents how often they felt fearful or anxious; lonely; or that the older they get, the more useless they are, which provided an indication of negative affect. The responses were scored on a five-point Likert scale (excellent/always, good/often, so-so/sometimes, bad/rarely, and very bad/never) for each item, which resulted in summary scores from 6 to 30, with higher scores indicating greater SWB. Based on previous research (30), the responses to each item of SWB were categorized as always, often, and sometimes/rarely/never (excellent, good and so-so/bad/very bad).

#### 2.2.3 Covariates

Relevant covariates in this article included five aspects: sociodemographic characteristics, health status, lifestyle, financial status, and community psychological consulting services. Sociodemographic variables included age (65–79 years, ≥80 years), gender (male, female), place of residence (urban, rural), coresidence (with household member, alone, nursing home), marital status (currently married, divorced/widowed/single), and educational level (illiterate, primary school, junior high and above). Health status was measured by self-rated health (excellent/good, average, poor/very poor), activity of daily living (ADL) limitations (yes, no), and instrumental activities of daily living (IADL) limitations (yes, no). Only participants who were fully independent in ADL (bathing, dressing, toileting, indoor transferring, continence, and eating) or IADL (visiting neighbors, going shopping, cooking meals, doing laundry, walking 1 km, carrying 5 kg weight, crouching and standing three times and taking public transportation) were deemed to have no I/ADL limitations (31, 32). Lifestyle variables included whether the respondents currently smoked (yes, no), and drank alcohol (yes, no), exercised regularly (yes, no), participated in outdoor activities (yes, no), and participated in social activities (yes, no). Financial status was assessed by asking the respondents to rate their family financial status (very rich/rich, average, and poor/very poor). They were also asked whether psychological consulting services were available in their community (yes, no).

### 2.3 Statistical analysis

Latent class analysis (LCA) is a statistical method used to discover and understand distinct latent classes based on individuals’ responses to a set of observed variables. The goal of LCA in this study was to identify the optimal latent SWB classes and determine the probabilities of older adults without a close confidant belonging to each class. The optimal number of latent classes was obtained by increasing the number of identified SWB classes until no improvement was observed. Model fit was assessed by commonly used fit measures, including Akaike’s Information Criterion (AIC), the Bayesian Information Criterion (BIC), and the sample size-adjusted BIC (aBIC), for which relative minimum values indicated better model performance. In addition, the bootstrap likelihood ratio test (BLRT) and the Lo–Mendell–Rubin (LMR) were used to compare the differential distribution of the log likelihood ratio between nested models, and statistical significance (*p* < 0.05) implied that the k-class model was better than the k-1 model (33). Entropy was also used to assess the model fit, with values close to 1.0 indicating better class separation and values >0.8 indicating that individuals were precisely classified (34, 35).

Descriptive statistics were calculated by applying counts and frequencies for the categorical variables and mean or median for the continuous variables. The distribution differences of the entire sample against each SWB latent class were examined by the chi-squared test and nonparametric tests, as appropriate. Multiple logistic regression models were used to explore the relationship between the SWB latent classes and the observed variables, and class three was used as the reference group. LCA models and analysis were performed with Mplus (v8.3), and all other analyses were performed with SPSS 26.0 (SPSS Inc., Chicago, IL, United States). The statistical significance was set at *p* < 0.05 for all analyses.

## 3 Results

### 3.1 Sample characteristics

The characteristics of the study respondents are presented in Table 1. As expected, the sample population in this study had a significantly lower median SWB score than people who had someone to confide in (22.0 vs. 23.0, *p* < 0.001; Supplementary Table S1), and female respondents scored lower than male respondents (21.5 vs. 23.0, *p* = 0.035).

**Table 1:** The characteristics of the study respondents.

	Male (*n* = 138)	Female (*n* = 212)	Total sample (*n* = 350)	χ^2^/Z	*p*
Age				10.802	0.001
65–79	62 (44.9%)	59 (27.8%)	121 (34.6%)		
≥ 80	76 (55.1%)	153 (72.2%)	229 (65.4%)		
Place of residence				0.252	0.615
Urban (city/town)	75 (54.3%)	121 (57.1%)	196 (56.0%)		
Rural	63 (45.7%)	91 (42.9%)	154 (44.0%)		
Coresidence				0.209	0.901
With household member(s)	74 (54.0%)	112 (53.3%)	186 (53.6%)		
Alone	47 (34.3%)	70 (33.3%)	117 (33.7%)		
Nursing home	16 (11.7%)	28 (13.3%)	44 (12.7%)		
Marital status				17.435	< 0.001
Currently married	47 (34.8%)	32 (15.4%)	79 (23.0%)		
Divorced/widowed/single	88 (65.2%)	176 (84.6%)	264 (77.0%)		
Educational level				7.892	< 0.001
Illiterate	26 (22.2%)	129 (69.7%)	155 (51.3%)		
Primary school	52 (44.4%)	36 (19.5%)	88 (29.1%)		
Junior high and above	39 (33.3%)	20 (10.8%)	59 (19.5%)		
Self-rated health				0.333	0.739
Excellent/good	53 (38.4%)	82 (38.7%)	135 (38.6%)		
Average	58 (42.0%)	81 (38.2%)	139 (39.7%)		
Poor/very poor	27 (19.6%)	49 (23.1%)	76 (21.7%)		
ADL limitations				4.938	0.026
Yes	16 (11.6%)	44 (20.8%)	60 (17.1%)		
No	122 (88.4%)	168 (79.2%)	290 (82.9%)		
IADL limitations				33.430	< 0.001
Yes	71 (51.4%)	171 (80.7%)	242 (69.1%)		
No	67 (48.6%)	41 (19.3%)	108 (30.9%)		
Currently smoked				83.326	< 0.001
Yes	61 (44.9%)	9 (4.3%)	70 (20.3%)		
No	75 (55.1%)	199 (95.7%)	274 (79.7%)		
Currently drank alcohol				43.043	< 0.001
Yes	37 (27.4%)	7 (3.3%)	44 (12.7%)		
No	98 (72.6%)	204 (96.7%)	302 (87.3%)		
Exercised regularly				3.821	0.051
Yes	59 (43.1%)	68 (32.7%)	127 (36.8%)		
No	78 (56.9%)	140 (67.3%)	218 (63.2%)		
Participated in outdoor activities				0.139	0.709
Yes	88 (63.8%)	131 (61.8%)	219 (62.6%)		
No	50 (36.2%)	81 (38.2%)	131 (37.4%)		
Participated in social activities				0.373	0.541
Yes	20 (14.6%)	36 (17.1%)	55 (16.1%)		
No	117 (85.4%)	175 (82.9%)	292 (83.9%)		
Self-rated financial status				1.007	0.281
Very rich/rich	25 (18.8%)	30 (14.5%)	55 (16.2%)		
Average	79 (59.4%)	125 (60.4%)	204 (60.0%)		
Poor/very poor	29 (21.8%)	52 (25.1%)	81 (23.8%)		
Psychological consulting services in community				1.553	0.213
Yes	19 (14.2%)	40 (19.4%)	59 (17.4%)		
No	115 (85.8%)	166 (80.6%)	281 (82.6%)		
SWB score				2.104	0.035
Median (Q1, Q3)	23.0 (19,25)	21.5 (18.25,25)	22.0 (19,25)		
Range	8–30	7–30	7–30		

In the study sample, approximately two-thirds of the respondents were female and oldest-old (aged 80+ years), and more than half of them lived in urban areas (56.0%) and had no formal education (51.3%). However, relatively more male respondents were educated than female respondents. Remarkably, within the Chinese context, during the era in which the oldest-old individuals in our study lived, there was a distinct gender bias favoring males over females. This bias resulted in limited educational opportunities for women, shaping the demographic characteristics observed in our sample. It is worth noting that although more than half of the respondents (53.6%) lived with household members, they still reported that they had no confidant to share their private feelings and concerns. This may be because only 23.0% of the respondents were currently married, and they lived with adult children or other relatives who could not provide them with emotional support.

For health-related data, most of the respondents rated their general health as not poor (78.3%) and reported that they were independent in ADL (82.9%). However, most of them (69.1%) had difficulty performing at least one IADL, and the proportion of female respondents with IADL limitations was higher, which may be related to the older age of women in the sample population. Although most of the respondents did not currently smoke or drink, only a few of them (36.8%) exercised regularly. A total of 62.6% of the respondents reported that they participated in outdoor activities; however, the majority of them (83.9%) did not participate in social activities. Most of the older adults rated their financial status at the average level and above (76.2%), and only 17.4% of the respondents reported that psychological consulting services were available in their community.

### 3.2 LCA results of subjective well-being patterns

**Figure 1:**
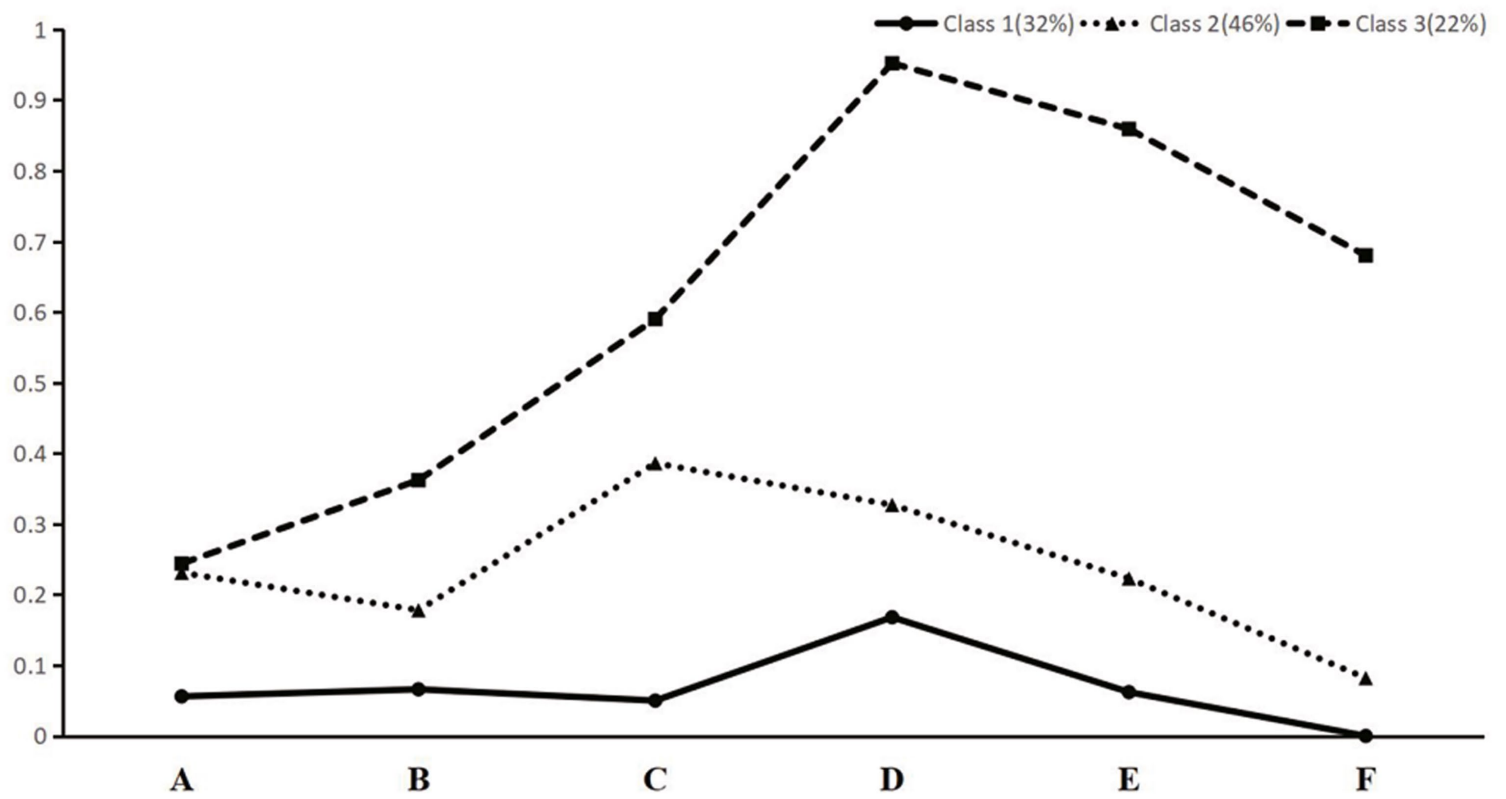
Conditional probability distribution for the three classes of SWB. **(A)** Life satisfaction (excellent); **(B)** look[ed] on the bright side of things (always); **(C)** [were] as happy as when they were young (always); **(D)** felt fearful or anxious (never); **(E)** felt lonely (never); and **(F)** self-perceived uselessness with age (never).

Table 2 shows the performance of latent class analysis (LCA) models with 1–5 classes. As Table 2 presented, the AIC was lowest when the number of classes was 5, the BIC was lowest when the number of classes was 3, and the aBIC was lowest when the number of classes was 4, however, the *p* value of the LMR was nonsignificant when the number of latent classes was four or five. Though the value of Entropy of three-class model was below 0.8 (0.756), it still beyond the criteria for good class separation cutoff point of 0.6 (36). Hence, the model with three groups was considered as the final model in this study. Figure 1 shows conditional probability distribution for the three classes of SWB, and Supplementary Table S2 shows the conditional probability of SWB indicators among three classes in detail (Supplementary Table S2). Using latent class analysis, we identified three SWB patterns: class 1, “very low SWB” (*n* = 112, 32% of the sample); class 2, “medium-low SWB” (*n* = 161, 46% of the sample); and class 3, “low evaluative and high affective SWB” (*n* = 77, 22% of the sample).

**Table 2:** Performance of latent class analysis (LCA) models with one to five classes.

Classes	AIC	BIC	aBIC	Entropy	LMR	BLRT
1	4328.934	4375.229	4337.161	/	/	/
2	4057.463	4153.911	4074.602	0.732	0.0000	0.0000
3	3982.507	4129.108	4008.559	0.756	0.0184	0.0000
4	4162.865	4162.865	4001.075	0.787	1	0.0000
5	3965.944	4212.851	4009.821	0.796	1	0.3333

AIC, Akaike information criteria; BIC, Bayesian information criteria; aBIC, Sample-size adjusted BIC; LMR, *p* value for the Lo–Mendell–Rubin; and BLRT, *p* value for the bootstrapped likelihood ratio tests.

Table 3 shows the descriptive statistics and group differences among three patterns of SWB. Samples in the “very low SWB” class had the lowest probability of experiencing a high level of SWB across all six SWB items. In comparison to other classes, individuals in this group were predominantly female, older, less educated, had poorer self-rated health and more IADL limitations, did not participate in exercise and social activities, and had poorer financial status. People in class 2 experienced medium to low levels of SWB in each SWB item, so we labeled class two as “medium-low SWB.” Samples in this group had characteristics similar to the first group (e.g., mainly female, older, and less educated), but their self-rated health and financial status were better, and they tended to participate in social activities. Class 3 represented the smallest sample. Despite having lower satisfaction with life, respondents in this group were more likely to have the highest affective SWB compared to other classes, especially in the dimension of negative affect, so we labeled this class as “low evaluative and high affective SWB.” Compared to other classes, this class mainly included individuals who were younger, better educated, and physically and socially active, the proportion of individuals with poor self-rated health, IADL limitations, and poor financial status in this class was low, and the proportion of male respondents in this class was the highest (50.6%).

**Table 3:** Descriptive statistics and group differences among three patterns of SWB.

	Class 1	Class 2	Class 3	χ ^2^/H	*p*
Age				9.533	0.009
65–79	35 (31.0%)	48 (30.0%)	38 (49.4%)		
≥ 80	78 (69.0%)	112 (70.0%)	39 (50.6%)		
Gender				6.771	0.034
Male	36 (31.9%)	63 (39.4%)	39 (50.6%)		
Female	77 (68.1%)	97 (60.6%)	38 (49.4%)		
Place of residence				4.668	0.097
Urban (city/town)	54 (47.8%)	97 (60.6%)	45 (58.4%)		
Rural	59 (52.2%)	63 (39.4%)	32 (41.6%)		
Coresidence				11.942	0.018
With household member(s)	50 (45.0%)	90 (56.6%)	46 (59.7%)		
Alone	50 (45.0%)	43 (27.0%)	24 (31.2%)		
Nursing home	11 (9.9%)	26 (16.4%)	7 (9.1%)		
Marital status				5.493	0.064
Currently married	21 (19.1%)	33 (21.0%)	25 (32.9%)		
Divorced/widowed/single	89 (80.9%)	124 (79.0%)	51 (67.1%)		
Educational level				16.101	< 0.001
Illiterate	58 (64.4%)	74 (52.5%)	23 (32.4%)		
Primary school	21 (23.3%)	39 (27.7%)	28 (39.4%)		
Junior high and above	11 (12.2%)	28 (19.9%)	20 (28.2%)		
Self-rated health				42.847	< 0.001
Excellent/good	21 (18.6%)	76 (47.5%)	38 (49.4%)		
Average	46 (40.7%)	64 (40.0%)	29 (37.7%)		
Poor/very poor	46 (40.7%)	20 (12.5%)	10 (13.0%)		
ADL limitations				5.503	0.064
Yes	25 (22.1%)	28 (17.5%)	7 (9.1%)		
No	88 (77.9%)	132 (82.5%)	70 (90.9%)		
IADL limitations				23.233	< 0.001
Yes	86 (76.1%)	120 (75.0%)	36 (46.8%)		
No	27 (23.9%)	40 (25.0%)	41 (53.2%)		
Currently smoked				3.498	0.174
Yes	21 (18.8%)	28 (17.8%)	21 (28.0%)		
No	91 (81.3%)	129 (82.2%)	54 (72.0%)		
Currently drank alcohol				1.121	0.571
Yes	15 (13.4%)	17 (10.8%)	12 (15.6%)		
No	97 (86.6%)	140 (89.2%)	65 (84.4%)		
Exercised regularly				12.341	0.002
Yes	29 (26.1%)	59 (37.3%)	39 (51.3%)		
No	82 (73.9%)	99 (62.7%)	37 (48.7%)		
Participated in outdoor activities				5.635	0.060
Yes	63 (55.8%)	100 (62.5%)	56 (72.7%)		
No	50 (44.2%)	60 (37.5%)	21 (27.3%)		
Participated in social activities				11.725	0.003
Yes	7 (6.3%)	32 (20.0%)	17 (22.1%)		
No	104 (93.7%)	128 (80.0%)	60 (77.9%)		
Self-rated financial status				41.026	< 0.001
Very rich /rich	7 (6.4%)	27 (17.4%)	21 (28.0%)		
Average	56 (50.9%)	99 (63.9%)	49 (65.3%)		
Poor/very poor	47 (42.7%)	29 (18.7%)	5 (6.7%)		
Psychological consulting services in community				2.781	0.249
Yes	13 (12.3%)	31 (19.6%)	15 (19.7%)		
No	93 (87.7%)	127 (80.4%)	61 (80.3%)		
SWB score				255.316	< 0.001
Median (Q1, Q3)	17.0 (14.0, 19.0)	23.0 (21.0, 25.0)	26.0 (25.0, 29.0)		
Range	7–21	18–28	22–30		

### 3.3 Factors affecting the patterns of subjective well-being

Multiple logistic regression analysis was conducted to identify factors that affected the patterns of subjective well-being (Table 4). Respondents who were currently drinkers (yes vs. no, OR = 3.716, *p* = 0.043), rated their health as poor/very poor or average (poor/very poor vs. excellent/good, OR = 4.397, *p* = 0.016; average vs. excellent/good, OR = 3.503, *p* = 0.009), and had poor/very poor self-rated financial status (poor/very poor vs. very rich/rich, OR = 32.255, *p* < 0.001) were more likely to be in the “very low SWB” class than in the “low evaluative and high affective SWB” class, while older adults who actively participated in social activities (yes vs. no, OR = 0.252, *p* = 0.038) were less likely to be in the “very low SWB” class than in the “low evaluative and high affective SWB” class. Participants who had IADL limitations (yes vs. no, OR = 2.321, *p* = 0.043) and poor/very poor self-rated financial status (poor/very poor vs. very rich/rich, OR = 6.803, *p* = 0.008) were more likely to be in the “medium-low SWB” class than in the “low evaluative and high affective SWB” class.

**Table 4:** Results of multiple logistic regression analysis.

Variable	Class 1			Class 2		
	*B*	OR	*p*	*B*	OR	*p*
Age (Ref: 65–79)						
≥ 80	−0.447	0.640	0.399	−0.075	0.928	0.862
Gender (Ref: male)						
Female	0.686	1.986	0.230	−0.021	0.980	0.962
Place of residence (Ref: rural)						
Urban (city/town)	0.308	1.360	0.480	0.363	1.437	0.324
Coresidence (Ref: nursing home)						
With household member(s)	−0.992	0.371	0.196	−0.670	0.512	0.270
Alone	−0.375	0.687	0.641	−0.951	0.386	0.148
Marital status (Ref: currently married)						
Divorced/widowed/single	0.736	2.087	0.186	0.731	2.078	0.092
Educational level (Ref: junior high and above)						
Illiterate	0.653	1.922	0.325	0.633	1.883	0.229
Primary school	−0.102	0.903	0.873	0.285	1.330	0.551
Self-rated health (Ref: excellent/good)						
Poor/very poor	1.481	4.397	0.016	−0.542	0.581	0.319
Average	1.254	3.503	0.009	0.142	1.152	0.703
ADL limitations (Ref: no)						
Yes	0.622	1.863	0.304	−0.011	0.989	0.984
IADL limitations (Ref: no)						
Yes	0.502	1.652	0.350	0.842	2.321	0.043
Currently smoked (Ref: no)						
Yes	0.519	1.681	0.382	−0.232	0.793	0.634
Currently drank alcohol (Ref: no)						
Yes	1.313	3.716	0.043	0.310	1.364	0.579
Exercised regularly (Ref: no)						
Yes	−0.823	0.439	0.066	−0.235	0.791	0.508
Participated in outdoor activities (Ref: no)						
Yes	−0.086	0.918	0.854	−0.111	0.895	0.786
Participated in social activities (Ref: no)						
Yes	−1.380	0.252	0.038	−0.143	0.867	0.749
Self-rated financial status (Ref: very rich/rich)						
Poor/very poor	3.474	32.255	< 0.001	1.917	6.803	0.008
Average	1.294	3.648	0.075	0.417	1.518	0.328
Psychological consulting services in community (Ref: no)						
Yes	−0.343	0.710	0.590	0.053	1.054	0.909

“Class 3” was used as the reference group.

## 4 Discussion

Previous studies have generated important insights into the association between confidant relationships and older adults’ emotional well-being (14, 15, 37). However, it is unknown whether there are different SWB subtypes among older adults without close confidants. What are the different subtype profiles of these people and their determinants, if any? In the current work, we applied the LCA method to examine the heterogeneity of SWB within older adults without close confidants and associated factors rather than treating them as a whole without distinction or focusing on their well-being at the mean. Moreover, we explored potential gender differences in the latent classes of SWB among socially isolated older adults.

The results showed that older respondents who had no one to confide in scored significantly lower in SWB than those who did have, which was consistent with previous researches showing that confidant availability was linked to higher SWB (14, 38, 39). These findings not only reinforce the established research but also spotlight the critical need for developing and implementing mechanisms to proactively identify lonely older adults within the community. Addressing this often-neglected and under-researched segment of the older population is essential for comprehensive community health strategies. By applying the clustering method, our study identified three SWB patterns among older people who lack a close confidant: “very low SWB,” “medium-low SWB,” and “low evaluative and high affective SWB.” We found that “medium-low SWB” represented the largest class, while the proportion of older people in the “low evaluative and high affective SWB” class was the smallest of the three classes. In particular, our study found that about one-third of older respondents were in the group of very low SWB, indicating that they are missing out on a happy life. In addition, our research found that the three classes differed significantly in SWB scores: older adults in the “very low SWB” class had a median SWB score of only 17.0 (14.0, 19.0), while the “low evaluative and high affective SWB” class scored much higher (26.0). Moreover, we found large differences in the level of negative affect among the three classes and smaller variations in life satisfaction and positive affect. Previous research has demonstrated that harmonious relationships with others are valued more in collectivistic cultures compared to individualistic cultures, which may partially explain the lower life satisfaction in all three classes (40). Given the heterogeneity in the overall state and three measures of SWB in older adults without confidants, this study suggests the need to explore the latent SWB types in this vulnerable group to facilitate more effective and targeted intervention.

Furthermore, the bivariate analysis revealed that a significantly higher proportion of female respondents than male respondents were in the “very low SWB” class. However, when considering the impact of multiple factors on SWB in a multinomial logit model, gender was not significantly associated with respondents’ SWB patterns. The result may be because compared to their male counterparts, the proportion of female respondents who were the oldest old, less educated, divorced/widowed/single, and had I/ADL limitations was higher, which may weaken the direct relationship between gender and subjective well-being.

The same situation has also occurred with factors such as age, coresidence and educational level. This implies that the interplay between different sociodemographic characteristics is intricate, and the effect of any single factor on subjective well-being can be obscured when the combined influence of several factors is considered. The lack of statistical significance in the multinomial logit model indicates that the predictive power of age, co-residence, and educational level on SWB may be contingent on other variables in the model, highlighting the multifaceted nature of subjective well-being determinants.

We used a multiple logistic regression analysis to identify factors associated with SWB patterns and considered “low evaluative and high affective SWB” as the reference group. The study revealed that membership in each of these classes was influenced by a different array of factors. Respondents who did not participate in social activities, had self-rated poor/very poor health, currently drank alcohol and rated their financial status as poor/very poor were more likely to report very low SWB, while those who had IADL limitations and rated their financial status as poor/very poor were more likely to report medium-low SWB.

It is worth mentioning that compared to older people who did not participate in social activities; we found that those who participated in social activities were less likely to be in the “very low SWB” class than in the “low evaluative and high affective SWB” class. However, this correlation did not exist for participation in outdoor activities, which reflects the unique sense of benefit to older people from social participation. Unlike outdoor activities, for social participation, the individual must be involved in an activity that provides contact with others in society or the community (41, 42). Our study found that older people who lacked confidant relationships might benefit from participating in social activities and exhibit better well-being, especially affective well-being. Similar studies have demonstrated a positive association between social participation and physical and mental health and well-being (43–46). This finding further validates previous research stating that social participation should be proposed as a mean and intervention goal of health professionals to reduce the adverse health effects of social isolation and loneliness in older adults (46, 47). Furthermore, increasing attention should be given to the social determinants of mental health and well-being in later life.

Respondents who rated their health as not good were more likely to be in the “very low SWB” class than those who rated their health as excellent or good. This result was consistent with previous studies. The relevance between self-rated health and the well-being of older adults has been demonstrated in different cultural backgrounds (30, 48, 49). In fact, some researchers suggest that self-rated health is an important, reliable and modifiable indicator of an individual’s physical, psychological, and social well-being (48). In a prospective study of 719,671 UK women, the researchers demonstrated that poor self-rated health was strongly associated with self-reported unhappiness (30). On the other hand, well-being was considered a protective factor in health maintenance at older ages (50). Hence, we assume that self-rated health and SWB also interact in our sample population. However, we could not determine a causal inference in this cross-sectional study. Respondents who reported drinking alcohol were more likely to be in the “very low SWB” class compared to those who did not drink alcohol. Numerous studies have confirmed a positive association between a healthy lifestyle and SWB (28, 51). Previous research has found that heavy alcohol consumption might cause adverse SWB in middle-aged men (52). Conversely, unhappiness might cause people to engage in unhealthy behavior and lifestyles (53). Unfortunately, the data we used do not indicate the amount of alcohol consumed by the respondents. However, to make more precise inferences in a sample of older adults who lack close confidants, it would be reasonable to consider in future studies whether alcohol consumption is excessive.

Previous reports have established the effects of subjective financial satisfaction even more than objective income on well-being (54–56). This may be because subjective income adequacy is often influenced by social comparison and expectations (56, 57). Relevant literature notes that income can not only directly improve people’s sense of well-being but also indirectly improve their SWB by increasing their social capital (58). Our results with a sample of socially isolated older adults are aligned with these previous studies. Respondents who rated their financial status as poor were more likely to be in the “very low SWB” class or “medium-low SWB” class than in the “low evaluative and high affective SWB” class. In addition to improving absolute income, the results have implications for strategies to reduce older adults’ relative deprivation to effectively enhance their SWB.

Our results showed that participants with IADL limitations were more likely to be in the “medium-low SWB” class than in the “low evaluative and high affective SWB” class, which means that whether the respondents had IADL limitations mainly affected their affective well-being rather than their life satisfaction. IADL, such as visiting neighbors, shopping, and cooking, represent complex activities that require a higher level of autonomy and cognitive ability (59, 60). It is not surprising that impairment of IADL functions may result in reduced affective well-being. This finding is consistent with the literature that shows that functional deficits in IADL are directly associated with depression (61). However, evidence of the relationship between IADL limitations and life satisfaction is inconsistent. Although this may not be entirely correct, scholars who hold the top-down perspective regard life satisfaction as a function of personality and other stable traits (62). Using growth curve modeling, Cheng KJG et al. revealed that I/ADL limitations adversely affected life satisfaction among middle-aged and older adults (63). In this study, we observed a lack of correlation between ADL limitations and SWB, which is inconsistent with results from other studies (61, 63–65). This could be a consequence of only 17.1% of the sample having ADL limitations and only 9.1% of them being assigned to the “low evaluative and high affective SWB” class. Furthermore, a smaller sample size may make it more difficult to reach statistical significance.

The strength of the current study is that, to our knowledge, this is the first study to apply LCA to investigate SWB patterns among older adults without close confidants rather than relying solely on their mean SWB score. In addition, the present study reveals predictors of very low SWB among socially isolated older adults, which may enable tailored interventions in the future. Furthermore, our study adopts a multidimensional approach to research the impact of confidant relationships on respondents’ SWB, whereas existing work has typically focused on single domains, such as depression, anxiety, and life satisfaction. However, it should be noted that there are some limitations in the current study. First, the cross-sectional design could only reveal correlations, and the nature of this methodology may not be applicable for drawing causal conclusions between the unavailability of confidants and older adults’ SWB. Thus, longitudinal research is needed to explore causality. Second, the explanatory variables investigated in this study may not be perfect indicators of older adults’ SWB because the CLHLS was not specifically designed for our research.

## 5 Conclusion

Our study contributes to a better understanding of the SWB patterns in older men and women who lack a confidant. The findings of this study also have important policy implications. First, routine screening for older people with unmet needs for confidant relationships is advocated, similar to screening for other health risk factors, to identify those who are socially isolated and inform efforts to address their needs. Furthermore, future research is recommended to develop health care programs tailored to the specific SWB group of socially isolated older adults, particularly those who suffer from very low SWB. This vulnerable population has been largely unexplored in the current literature.

## Data availability statement

Publicly available datasets were analyzed in this study. This data can be found at: https://opendata.pku.edu.cn/file.xhtml?fileId=10356&version=2.1.

## Ethics statement

The studies involving humans were approved by the Ethics Committee of Peking University [IRB00001052-13074; The data used in this study came from the 2018 Chinese Longitudinal Healthy Longevity Survey (CLHLS), which was approved by the Ethics Committee of Peking University (IRB00001052-13074)]. The studies were conducted in accordance with the local legislation and institutional requirements. The participants provided their written informed consent to participate in this study.

## Author contributions

DM: Conceptualization, Data curation, Formal analysis, Writing – original draft, Writing – review & editing. CS: Conceptualization, Data curation, Formal analysis, Writing – original draft.
