# Supplementary material for: Subjective well-being patterns in older men and women without someone to confide in: a latent class analysis approach
Source: Front Public Health. 2024 Jan 5;11:1286627. doi: 10.3389/fpubh.2023.1286627 (PMC10796680; doi:10.3389/fpubh.2023.1286627)
Supplement: Supplementary file 1 [file Data_Sheet_1.pdf]

Supplementary Material

Figure S1: Flow chart of the study sample.

Table S1: Differences in median SWB score among respondents with and without a close confidant.

Table S2: Conditional probability of SWB indicators among three classes.

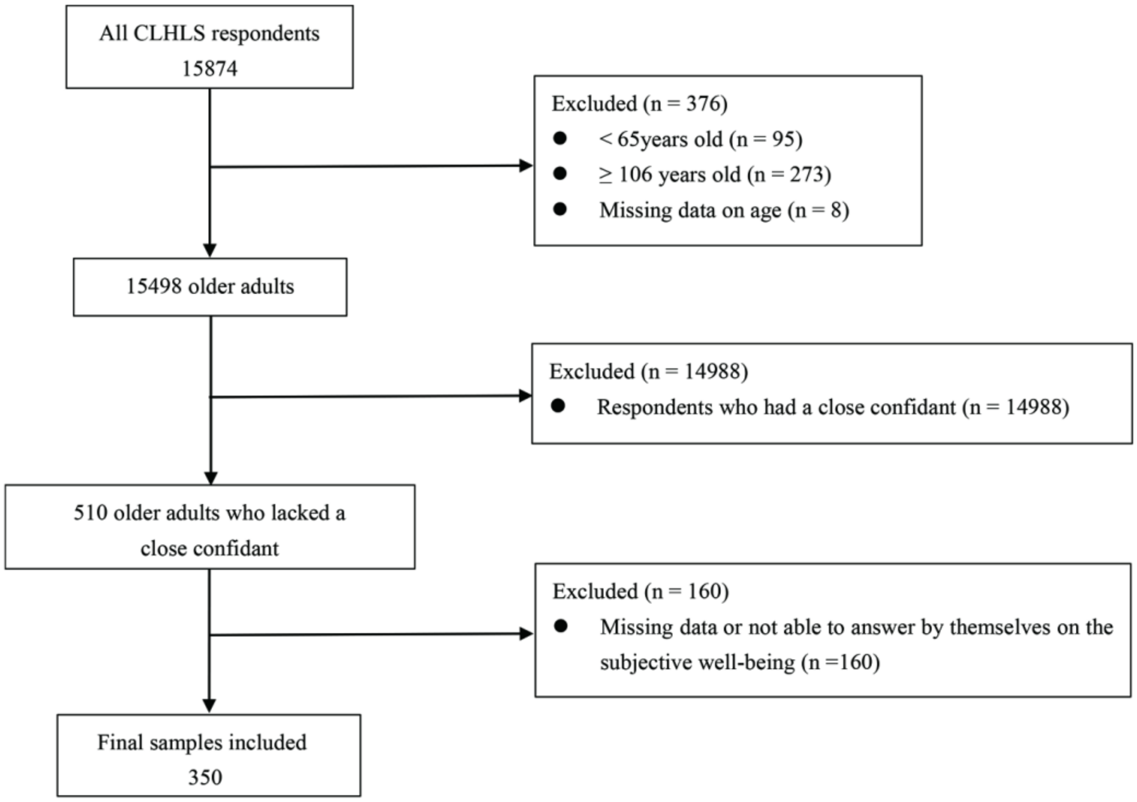

Fig. S1. Flow chart of the study sample.

Table S1: Differences in median SWB score among respondents with and without a close confidant.

SWB score	With a close confidant (n=12,274)	Without a close confidant (n=350)	Total (n=12,624)	Z	P
median (Q1, Q3)	23.0 (20,25)	22.0 (19,25)	23.0 (20,25)	3.798	<0.001
range	6-30	7-30	6-30		

Note. Excluding samples with missing SWB items, a total of 12624 older people

Table S2: Conditional probability of SWB indicators among three classes.

Items	Answers	Class 1	Class 2	Class 3
life satisfaction	excellent	0. 056	0. 231	0. 244
	good	0. 202	0. 5	0. 509
	so-so/bad/very bad	0. 742	0. 27	0. 247
look[ed] on the bright side of things	always	0. 066	0. 178	0. 362
	often	0. 364	0. 623	0. 638
	sometimes/rarely/never	0. 57	0. 199	0
[were] as happy as when they were young	always	0. 05	0. 386	0. 59
	often	0. 002	0. 246	0. 127
	sometimes/rarely/never	0. 948	0. 368	0. 283
felt fearful or anxious	never	0. 168	0. 327	0. 952
	rarely	0. 262	0. 526	0. 013
	sometimes/often/always	0. 57	0. 147	0. 035
felt lonely	never	0. 062	0. 223	0. 859
	rarely	0. 082	0. 466	0
	sometimes/often/always	0. 856	0. 311	0. 141
self-perceived uselessness with age	never	0	0. 082	0. 68
	rarely	0. 071	0. 482	0. 105
	sometimes/often/always	0. 929	0. 435	0. 215
